# Effects of dried distillers’ grains inclusion on performance, feed efficiency, carcass traits and health of feedlot lambs

**DOI:** 10.1007/s11250-026-05274-5

**Published:** 2026-08-03

**Authors:** Josiel Ferreira, Tiago do Prado Paim, Lucas Ferreira Gonçalves, Julia Stefeny dos Santos Silva, Divino Antonio Santana Lima, Guilherme Antonio Alves dos Santos

**Affiliations:** 1 Instituto Federal de Educação, Ciência e Tecnologia Goiano (IF Goiano), Campus Campos Belos, GO-118 Highway, Qd. 1-A, Lt. 1, Novo Horizonte District, Campos Belos, GO 73840-000 Brazil; 2 Empresa Brasileira de Pesquisa Agropecuária (EMBRAPA), Unidade Pesca e Aquicultura, Palmas, 77008-900 TO Brazil; 3 Instituto Federal de Educação, Ciência e Tecnologia Goiano (IF Goiano), Campus Iporá, Campus Iporá 75200-000 Brazil; 4 Universidade Estadual de Montes Claros (Unimontes), Departamento de Ciências Agrárias, Janaúba, MG 39448-581 Brazil; 5 Instituto Federal de Educação, Ciência e Tecnologia Goiano (IF Goiano), Campus Rio Verde, Goiás, GO 75901-970 Brazil

**Keywords:** Alternative feed, Distillers’ grains, Feed efficiency, Lamb production, Metabolic indicators

## Abstract

The use of agro-industrial by-products in ruminant diets has increased as a strategy to improve the sustainability of livestock production systems. This study evaluated the biological feasibility of replacing soybean meal with dried distillers’ grains with solubles (DDGS) at a practical dietary inclusion level in feedlot lamb diets. Twenty-four Santa Inês ewe lambs (27.08 ± 2.89 kg initial body weight) were assigned to a completely randomized design with two dietary treatments: a control diet without DDGS and a diet containing 6.27% DDGS (dry matter basis). The experimental period consisted of 21 days of dietary adaptation followed by 52 days of evaluation. Productive performance, feed intake, feed efficiency, in vivo carcass traits, parasitological indicators, hematological variables, and serum biochemical parameters were evaluated. Dietary inclusion of DDGS did not affect average daily gain, final body weight, body condition score, Longissimus muscle area, backfat thickness, dry matter intake, feed conversion ratio, or feed efficiency (*p* > 0.05). However, lambs fed the DDGS diet showed lower dry matter intake relative to body weight (*p* = 0.021). Parasitological indicators, including eggs per gram of feces, Famacha© score, and packed cell volume, were not affected by dietary treatment. Most hematological and serum biochemical variables were also unaffected. Lambs fed the DDGS diet exhibited lower leukocyte and neutrophil counts (*p* < 0.05), whereas serum urea concentration was higher (*p* = 0.035), likely reflecting the higher dietary crude protein content. Overall, replacing soybean meal with DDGS at a commercially relevant inclusion level maintained productive performance, carcass characteristics, and the evaluated hematological and serum biochemical indicators in feedlot lambs under intensive tropical production conditions.

**Graphical Abstract:**

**Figure:**
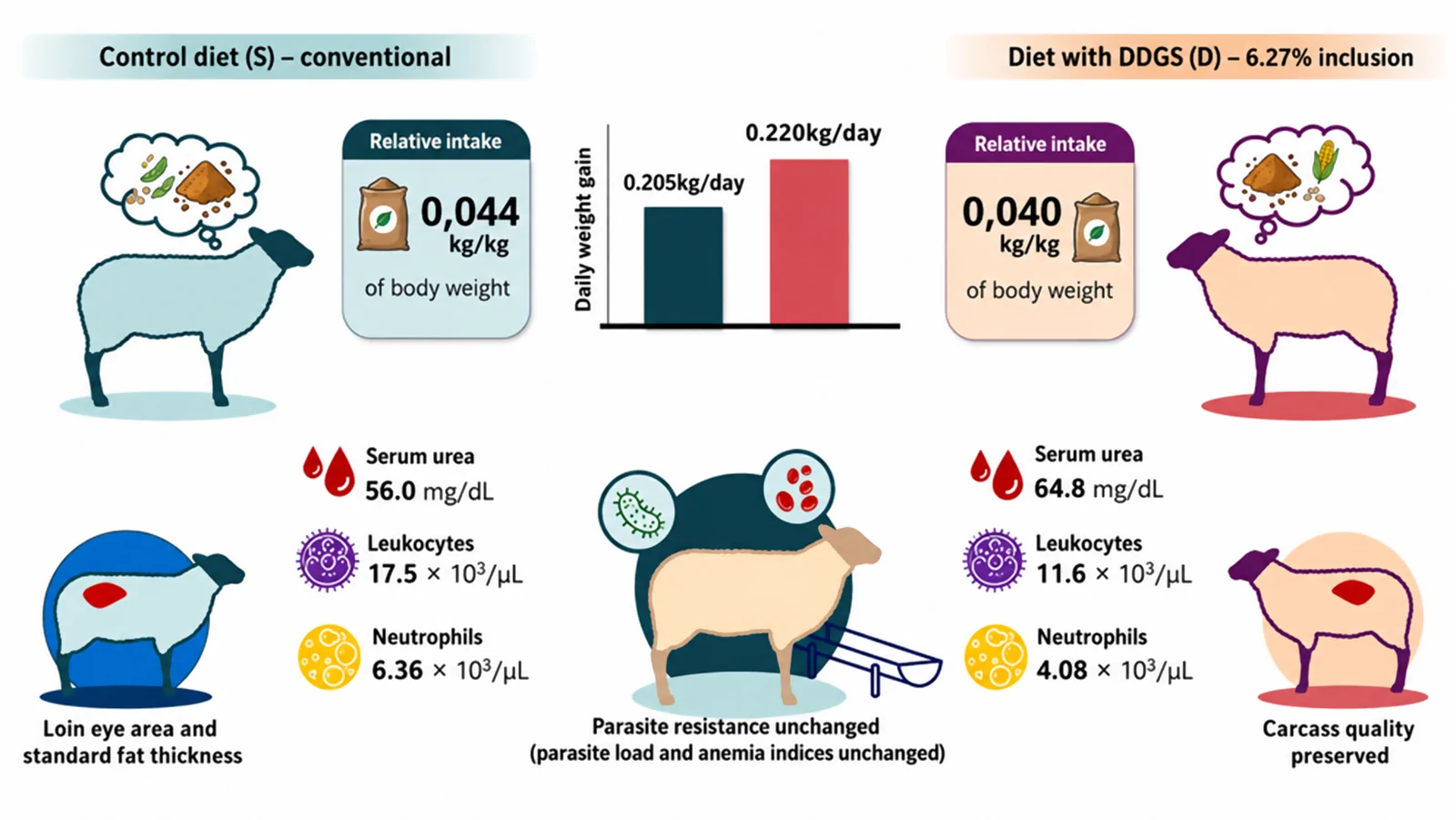

## Introduction

Feed supplementation is a key component of ruminant production systems, directly affecting animal performance, feed efficiency, and economic returns. The use of agro-industrial by-products as alternative feed ingredients has increased in recent years, driven by the need to reduce feeding costs and improve the sustainability of livestock systems (Landim et al. 2025). Among these alternatives, dried distillers’ grains with solubles (DDGS), a co-product of the ethanol industry, have attracted attention due to their high protein and energy content, wide availability, and competitive cost (Sorensen et al. 2021; Pannell et al. 2022; Reddy et al. 2024).

Previous studies have demonstrated that DDGS can successfully replace conventional protein sources in ruminant diets, although animal responses vary according to inclusion level, diet composition, and production system. In beef cattle, moderate DDGS inclusion generally maintains or improves growth performance and feed efficiency (Hollmann et al. 2011; Griffin et al. 2012). Similarly, studies with sheep and goats have reported that partial replacement of soybean meal or cottonseed meal by DDGS does not impair average daily gain, carcass characteristics, or metabolic health when moderate inclusion levels are adopted (Ale et al. 2022; Quadros and Kerth 2022; Reddy et al. 2021, 2024). However, increasing DDGS inclusion may reduce dry matter intake, alter rumen development, and modify nitrogen metabolism, particularly when high dietary concentrations are used (Avila-Stagno et al. 2013; Chelkapally et al. 2023; dos Santos et al. 2024). These findings indicate that the biological responses to DDGS depend not only on its inclusion rate but also on the nutritional characteristics of the basal diet and the production system.

The inclusion of DDGS has been investigated as an alternative protein source because of its nutritional value and wide availability (Reddy et al. 2021). Furthermore, the incorporation of ethanol by-products into ruminant diets highlights the need to evaluate metabolic and health-related indicators, including blood biochemical and hematological parameters (Ale et al. 2022; Gite et al. 2024), particularly under intensive feeding conditions. Although several studies have evaluated DDGS in sheep diets, most have investigated different production systems, ingredients, and inclusion rates. The present study was designed to evaluate the practical replacement of soybean meal by DDGS at a single commercially relevant inclusion level under tropical feedlot conditions. Therefore, this study was not intended to establish a dose-response relationship or determine the optimal inclusion level, but rather to assess the biological feasibility of this dietary substitution under intensive tropical production conditions. Feed efficiency is a central factor determining the profitability of feedlot operations. Traditional indices such as feed conversion ratio (FCR) and feed efficiency (FE), provide complementary information on the animal’s ability to convert feed into body weight gain (Sartori et al. 2024). Highly digestible protein and energy sources may improve these indices by enhancing nutrient utilization efficiency (Ellison et al. 2022; Chelkapally et al. 2023), suggesting that DDGS could positively influence feed efficiency in lambs.

Therefore, the objective of this study was to evaluate the biological feasibility of replacing soybean meal with DDGS at a commercially relevant inclusion level in feedlot lamb diets under tropical conditions. We hypothesized that this dietary substitution would maintain animal performance, carcass traits, and physiological homeostasis without adverse effects on feed efficiency or health indicators.

## Materials and methods

### Ethical approval, animals, and housing

All procedures involving animals were approved by the Animal Ethics Committee of the *Instituto Federal Goiano* (protocol no. 1827080323). The experiment was conducted at the Sheep Feedlot Facility of the *Instituto Federal Goiano* – *Campus Rio Verde*, Goiás, Brazil (17°48′53.1″ S, 50°53′52.6″ W; altitude 701 m).

The trial was carried out in a covered feedlot barn with a ceiling height of approximately 4 m. Lambs were housed in pairs in collective pens measuring 2 × 5 m (10 m^2^), resulting in approximately 5 m² per animal. Pens had concrete floors and no bedding material was used. Each pen was equipped with two individual feeders and one automatic drinker, allowing *ad libitum* access to water throughout the experimental period. Standard health management and daily animal inspections were conducted throughout the trial.

Twenty-four Santa Inês ewe lambs, approximately six months old, with an initial body weight of 27.08 ± 2.89 kg, were used. Prior to the trial, all animals underwent clinical evaluation and were considered healthy. Animals were also evaluated for gastrointestinal parasite infection to ensure similar initial health status among treatments.

### Experimental design

The experiment was conducted as a completely randomized design with two dietary treatments and six replicate pens per treatment (12 animals per treatment). Treatments consisted of: (1) a control diet without DDGS and (2) a diet containing 6.27% DDGS (dry matter basis), replacing soybean meal. The experimental diets contained 14.99 and 17.78% crude protein and 3.07 and 2.86 Mcal kg⁻¹ metabolizable energy for the control and DDGS diets, respectively (Table 1).

**Table 1:** Chemical composition of the corn silage and dried distillers grains dried distillers grains with solubles (DDGS)

Variable (unit)	Corn silage	DDGS
Humidity (%)	69.05	9.96
Dry matter (%)	30.95	90.04
Crude protein (%DM)	8.40	33.46
Soluble protein (%DM)	65.41	-
Digestible protein (%DM)	8,01	-
Acid-detergent insoluble protein (%DM)	0.38	1.52
Acid-detergent insoluble protein (%CP)	4.58	-
Neutral-detergent insoluble protein (%DM)	0.93	2.12
Neutral detergent fiber (%DM)	31.22	31.70
Acid-detergent fiber (%DM)	49.57	15.36
Neutral detergent fiber in organic matter (%DM)	47.81	-
Crude fiber (%DM)	-	7.93
Lignin (% DM)	5.55	4.04
Lignin (% NDF)	11.2	12.76
Starch (% DM)	20.01	5.00
Starch (% NFC)	57.36	21.44
Ethereal Extract (%DM)	2.78	7.16
Ash (%DM)	5.3	6.46
Non-fibrous carbohydrates (%DM)	34.88	23.34
Total digestible nutrients (%DM)	59.70	75.49

**Table 2:** Percentage of the ingredients e chemical composition of the diets

Ingredients (%)	Treatments	
	S	D
Corn	48.46	46.79
Soybean Hulls	31.33	31.32
Cottonseed	13.07	11.08
Kernel	3.96	3.96
Soybean Meal	2.64	-
Limestone	0.54	0.56
DDGS	-	6.27
Chemical composition (unit)		
Dry matter (%)	92.98	92.87
Moisture (%)	7.02	7.13
Neutral detergent fiber (%DM)	19.81	16.46
Acid detergent fiber (%DM)	27.33	24.88
Lignin (%DM)	2.35	2.34
Starch (%DM)	33.29	30.59
Crude fiber (%DM)	13.88	11.67
Lignin (%NDF)	8.62	9.42
Starch (%NFC)	70.30	66.81
Crude protein (%DM)	14.99	17.78
Acid detergent insoluble protein (%DM)	0.76	0.61
Neutral detergent insoluble protein (%DM)	1.21	1.1
Ether extract (%DM)	4.28	5.08
Ash (%DM)	7.35	7.58
Non-fibrous carbohydrate (%DM)	47.36	45.78
Total digestible nutrients (%DM)	79.49	79.12
Digestible energy (Mcal/kg)	3.50	3.26
Metabolizable energy (Mcal/kg)	3.07	2.86
Net energy for gain (Mcal/kg)	1.28	1.2
Net energy for maintenance (Mcal/kg)	1.93	1.8

Dried distillers grains with solubles (DDGS); S: control diet; D: diet containing 6.27% of dried distillers grains (30% crude protein)

Lambs were randomly assigned to treatments. Animals were housed in pairs, with each pen (*n* = 12) considered the experimental unit for feed intake and productive performance variables. Individual animals were considered the observational units for repeated physiological, hematological, and serum biochemical measurements. To minimize potential environmental variation within the feedlot facility, adjacent pens received alternating dietary treatments. The experimental period lasted 52 days, following a 21-day adaptation period.

During the adaptation phase, corn silage was used as the roughage source. The diet was gradually transitioned from a 70:30 concentrate-to-roughage ratio to the experimental 100% concentrate diet by increasing concentrate inclusion by approximately 10% units per week (80:20 during the second week and 90:10 during the third week). The chemical composition of the corn silage and DDGS is presented in Table 1. A 100% concentrate diet was selected to simulate intensive commercial feedlot systems commonly adopted during the finishing phase of lamb production in Brazil and to maximize the evaluation of DDGS as a substitute for soybean meal under high-concentrate feeding conditions.

Diets (Table 2) were formulated to meet the nutrient requirements of growing lambs with an expected body weight of 35 kg and an average daily gain of 300 g day⁻¹, according to NRC (2007). Feed was offered twice daily (08:00 and 16:00 h), with ad libitum access to water.

### Feed intake and performance

Feed intake was recorded daily by weighing the feed offered and refusals. Dry matter intake per animal (DMI) was calculated based on the dry matter content of feeds and orts. Dry matter intake relative to body weight (DMI/BW; %BW) was calculated using body weight measurements obtained at each evaluation period.

Lambs were weighed at the beginning of the experiment and at regular intervals (14 days) throughout the experimental period. Average daily gain (ADG) was estimated by linear regression of body weight over time for each experimental unit, using the slope of the regression equation as the estimate of daily weight gain. Final body weight (FBW) was recorded at the end of the experimental period. Body condition score (BCS) was assessed using a 1-to-5 scale, according to Machado et al. (2008). Therefore, performance variables evaluated were ADG, FBW, BCS, DMI per animal (kg day^− 1^), and DMI relative to body weight (% BW).

### Feed efficiency

Feed efficiency was evaluated using feed efficiency (FE) and feed conversion ratio (FCR) obtained using the formula used by Sartori et al. (2024). Feed efficiency was calculated as the ratio between ADG and DMI, whereas feed conversion ratio was calculated as the ratio between DMI and ADG. Both variables were calculated for each evaluation period and analyzed as repeated measures.

### In vivo carcass traits

In vivo carcass traits were evaluated at four different moments during the experiment using real-time ultrasound. *Longissimus* muscle area (LMA; cm^2^) and backfat thickness (BT; mm) were measured between the 12th and 13th ribs using a KX5600 full digital B-mode ultrasound scanner equipped with a 3.5 MHz linear transducer. Images were analyzed using ImageJ (Schindelin et al. 2012) to estimate muscle size and subcutaneous fat deposition.

### Fecal analysis and anemia indicators

Fecal samples were collected directly from the rectum of each animal at four time points throughout the experimental period. Approximately 10 g of feces were placed into individually identified plastic bags, stored in insulated boxes with ice packs, and transported to the laboratory. Samples were refrigerated at 4 °C and processed within 48 h. Parasitological examination focused on the egg’s detection and quantification of gastrointestinal nematodes belonging to the superfamily *Strongyloidea*, which includes hematophagous species such as *Haemonchus contortus*. The results are expressed as eggs per gram of feces (EPG), using the modified McMaster technique (Gordon and Whitlock 1939).

The Famacha© score was assessed simultaneously at each sampling time to estimate the degree of anemia by evaluating the color of the ocular mucous membranes using a standardized five-point chart, where score 1 indicates non-anemic animals and score 5 indicates severe anemia, following Van Wyk and Bath (2002). Packed cell volume was determined at each sampling time using the microhematocrit method, which consists of centrifugation of whole blood in capillary tubes followed by measurement of the erythrocyte column relative to total blood volume. This method is widely used for packed cell volume (PCV) determination and anemia assessment (Mondal and Zubair 2025).

### Hematological and serum biochemical analyses

Blood samples were collected by jugular venipuncture at the beginning and at the end of the experimental period using vacuum tubes with and without anticoagulants. Samples collected with anticoagulant were used for hematological analyses, whereas samples without anticoagulant were centrifuged at 3,000 × g for 10 min to obtain serum for biochemical determinations.

Complete blood counts were performed using an automatic hematology analyzer (Counter XS20, Wiener Lab Group). The erythrogram included red blood cell count, hemoglobin, packed cell volume, mean corpuscular volume, mean corpuscular hemoglobin, mean corpuscular hemoglobin concentration, and erythroblasts (cells observed during differential count). The leukogram included total leukocyte count and differential leukocyte counts, expressed as percentages of segmented neutrophils, eosinophils, lymphocytes, and monocytes. Plasma total protein was also determined.

Serum biochemical analyses were performed using an automatic biochemical analyzer (Vet RT-1904CV) and included parameters related to energy metabolism (glucose), lipid metabolism (triglycerides and total cholesterol), liver function (alanine aminotransferase [ALT] and aspartate aminotransferase [AST]), protein profile (total proteins and albumin), and renal function (urea and creatinine).

### Statistical analysis

All statistical analyses were performed using the software R (version 4.3.2; R Core Team 2023). Linear mixed-effects models were fitted using the *lme4* package (Bates et al. 2015), and estimated marginal means were obtained using the *emmeans* package (Lenth and Piaskowski 2025). Statistical significance was declared at *p* ≤ 0.05.

Variables related to animal performance, including average daily gain ADG, FBW, BCS, REA, and BT, were analyzed using linear mixed models with repeated measures. The models included treatment, evaluation day, and their interaction as fixed effects, with initial body weight (IBW) included as a covariate. Animal was included as a random effect to account for repeated measurements on the same individual over time.

DMI was analyzed using a linear mixed model including treatment, evaluation day, and the treatment × day interaction as fixed effects, with pen included as a random effect to account for the experimental unit structure. Estimated marginal means were obtained using the *emmeans* package when significant effects were detected.

Health-related variables were analyzed using linear models including treatment as a fixed effect and the respective initial measurement as a covariate when applicable. Parasitological indicators, including EPG, Famacha© score, and PCV, were analyzed using linear mixed models with repeated measures. The models included treatment, evaluation day, and their interaction as fixed effects, with animal included as a random effect. Hematological and serum biochemical variables measured at the beginning and end of the experimental period were analyzed using linear mixed models including treatment and initial value as fixed effects and animal as a random effect. The initial value of the respective variable was included in the model as a covariate.

Model assumptions were evaluated by graphical inspection of residuals and by the Shapiro–Wilk test for normality. When required, data were log-transformed to meet model assumptions. Least-squares means were obtained and compared using Tukey’s adjustment for multiple comparisons.

## Results

### Productive performance, feed intake and carcass traits

**Fig. 1:**
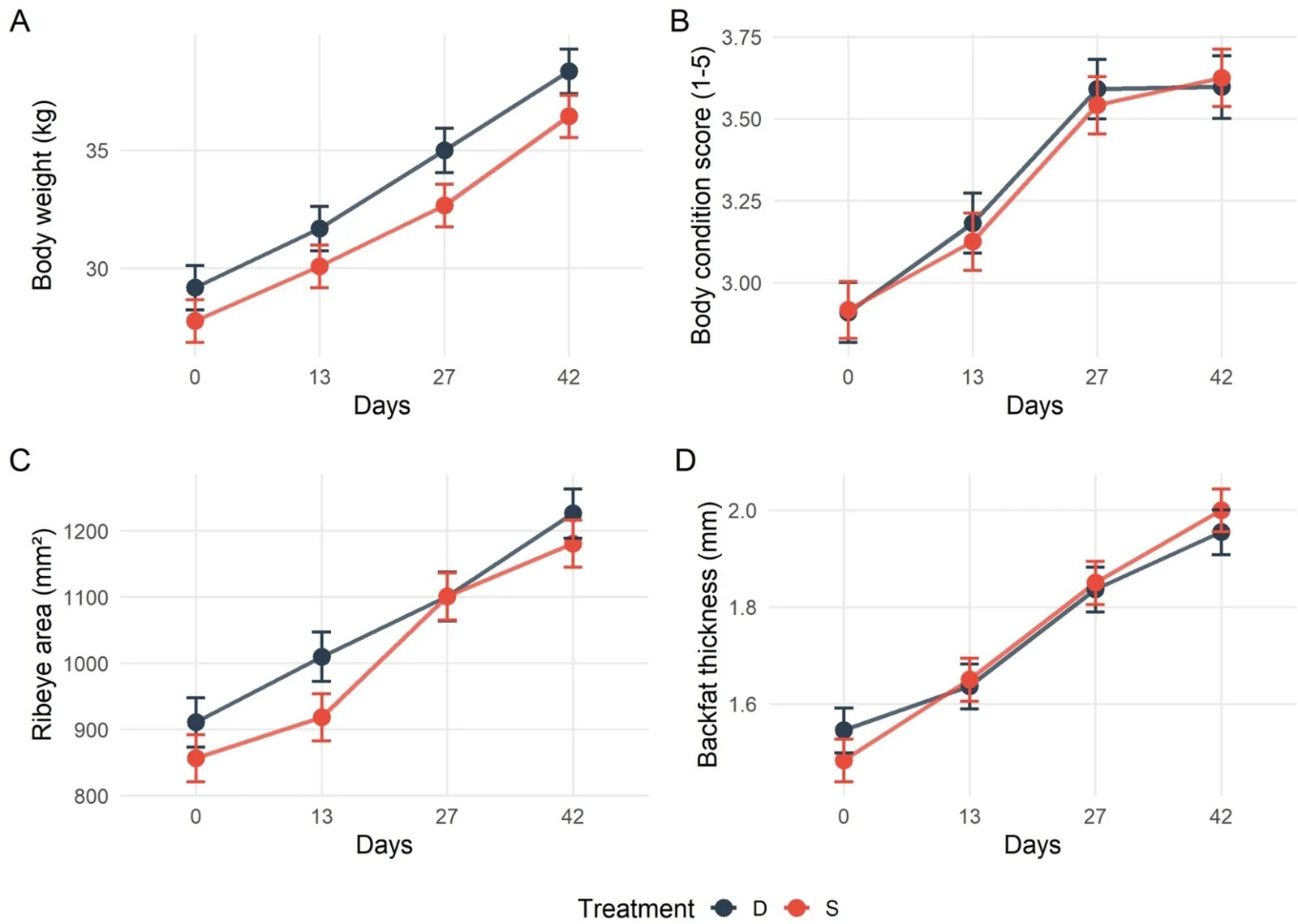
Productive performance, feed intake, feed efficiency, and in vivo carcass traits of feedlot lambs fed a control diet (S) or a diet containing dried distillers grains dried distillers grains with solubles (DDGS; D) throughout the experimental period. Values represent least squares means ± standard error

Dietary inclusion of DDGS did not affect ADG, FBW, BCS, LMA, BT, DMI, FCR, or FE (*p* > 0.05; Table 3; Fig. 1). However, lambs fed the control diet (S) showed greater DMI relative to body weight compared to those fed the DDGS-based diet (D) (0.044 vs. 0.040 kg/kg BW; *p* = 0.021). A significant effect of evaluation days was observed for FBW, BCS, LMA, BT, DMI, FCR, and FE (*p* < 0.05), indicating progressive changes throughout the experimental period (Fig. 1). No treatment × days interaction was detected for any performance variable (*p* > 0.05). Figure 1 illustrates the temporal progression of performance and carcass traits, demonstrating similar growth patterns between treatments over time.

**Table 3:** Least squares means and analysis of variance for productive performance and feed efficiency of lambs fed control (S) and dried distillers grains with solubles (DDGS)-based diets (D)

Variable (unit)				*p*-values according to different effects		
	D	S	SEM	Treatment	Days	Treatment × Days
ADG (kg day^− 1^)	0.220	0.205	0.012	0.240	–	–
FBW (kg)	37.9	36.8	0.816	0.212	< 0.001	0.160
BCS (1–5)	3.59	3.64	0.122	0.811	< 0.001	0.953
LEA (mm²)	1220	1186	51	0.358	< 0.001	0.360
BT (mm)	1.95	2.01	0.063	0.751	< 0.001	0.551
DMI (kg day^− 1^)	1.365	1.412	0.0568	0.400	< 0.001	0.240
DMI (kg kg^− 1^ BW)	0.040ᵇ	0.044ᵃ	0.0018	0.021	< 0.001	0.814
FCR (kg DM kg^− 1^ gain)	6.90	7.06	0.63	0.798	< 0.001	0.340
FE (kg gain kg^− 1^ DM)	0.154	0.145	0.009	0.404	0.001	0.680

ADG = average daily gain; FBW = final body weight; BCS = body condition score; LEA = *Longissimus* muscle area; BT = subcutaneous fat thickness; DMI = dry matter intake; FCR = feed conversion ratio; FE = feed efficiency

Values are presented as least squares means ± standard error of the mean (SEM)

*p*-values refer to the fixed effect of treatment, days and interaction (treatment × days)

Statistical significance was set at *p* ≤ 0.05

“–” indicates that the effect was not tested

### Parasitological indicators and anemia-related parameters

**Fig. 2:**
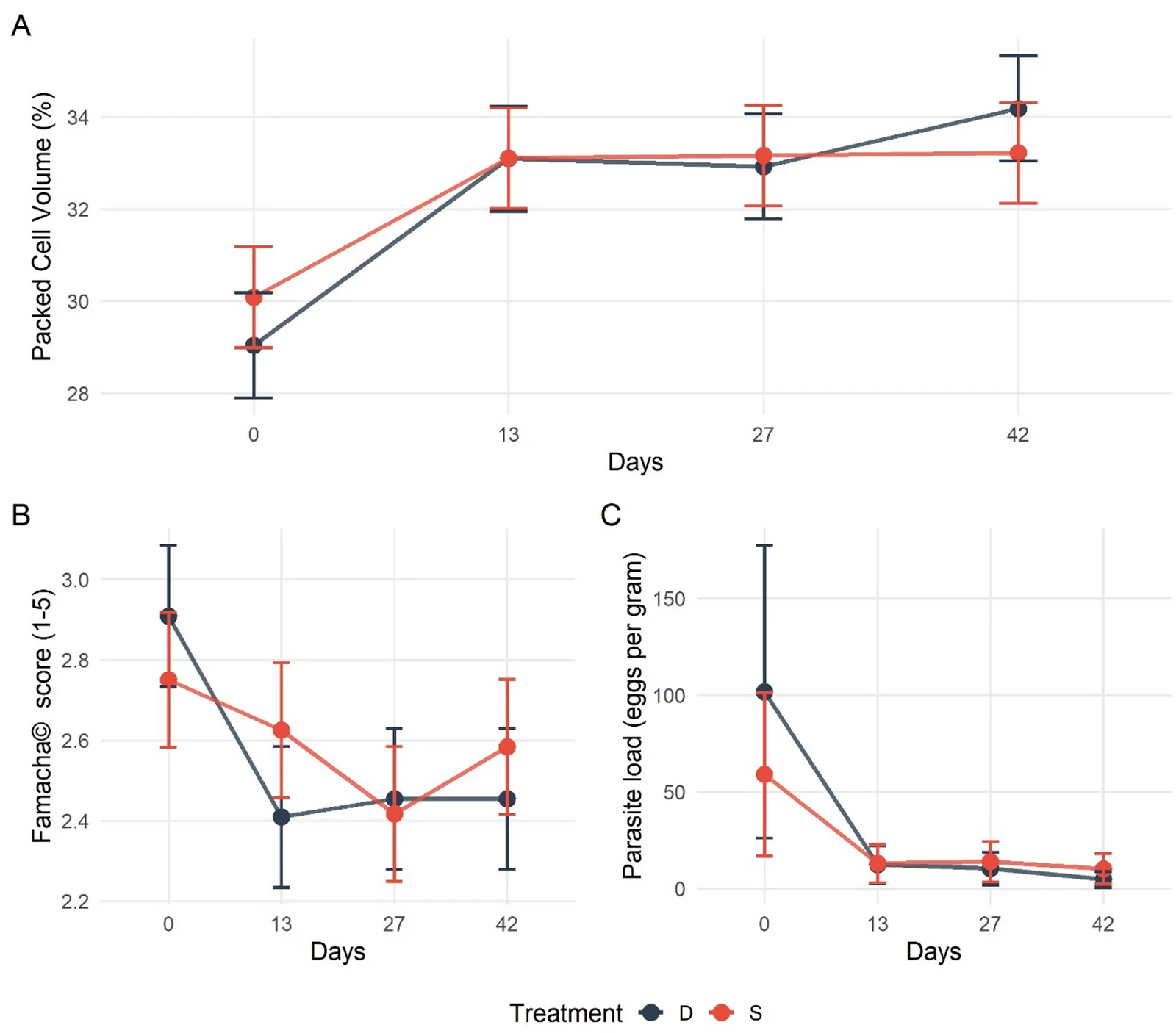
Parasitological burden and anemia-related indicators of feedlot lambs fed a control diet (S) or a diet containing dried distillers grains dried distillers grains with solubles (DDGS; D) during the experimental period. Values represent least squares means ± standard error

EPG, Famacha© score, and PCV were not affected by dietary treatment (*p* > 0.05; Fig. 2). A significant effect of evaluation moment was observed for EPG and PCV (*p* < 0.05), reflecting natural variation throughout the feeding period. No treatment × moment interaction was detected (*p* > 0.05). Figure 2 shows the dynamics of parasitological burden and anemia indicators over time, with similar patterns between treatments.

### Hematological parameters

No treatment effect was observed for erythrogram variables, including red blood cells, hemoglobin, packed cell volume, mean corpuscular volume, mean corpuscular hemoglobin, and mean corpuscular hemoglobin concentration (*p* > 0.05; Table 4). In contrast, lambs fed the DDGS-based diet presented lower total leukocyte counts (11.6 vs. 17.5 × 10^3^/µL; *p* = 0.008) and neutrophil counts (4.08 vs. 6.36 × 10^3^/µL; *p* = 0.031) compared to the control group. Eosinophils, lymphocytes, and monocytes were not affected (*p* > 0.05). Platelet counts were similar between treatments (*p* > 0.05).

**Table 4:** Least squares means and analysis of variance of hematological and serum biochemical parameters of lambs fed a control diet (S) and a dried distillers grains dried distillers grains with solubles (DDGS)-based diet (D)

Variable (unit)	D	S	SEM	Treatment *p*-value	Covariate *p*-value_i_
Erythrogram					
Red blood cells (×10^12^ L^− 1^)	10.8	10.2	0.68	0.539	0.164
Hemoglobin (g dL^− 1^)	12.3	11.8	0.22	0.099	0.049
Packed cell volume (%)	34.9	33.8	0.75	0.300	0.027
Mean corpuscular volume (fL)	32.8	32.4	1.66	0.889	0.733
Mean corpuscular hemoglobin (pg)	11.5	11.2	0.55	0.699	0.833
Mean corpuscular hemoglobin concentration (g dL^− 1^)	35.1	34.7	0.32	0.388	0.025*
Platelet profile					
Platelets (×10^3^ µL^− 1^)	1,154.8	1,114.7	204.0	0.899	0.842
Leukogram					
Leukocytes (×10^3^ µL^− 1^)	11.6b	17.5a	1.25	0.008**	0.003**
Neutrophils (×10^3^ µL^− 1^)	4.08b	6.36a	0.60	0.031*	0.005**
Eosinophils (×10^3^ µL^− 1^)	0.46	0.60	0.14	0.484	0.935
Lymphocytes (×10^3^ µL^− 1^)	6.08	7.79	1.11	0.345	0.415
Monocytes (×10^3^ µL^− 1^)	0.38	0.72	0.22	0.469	0.830
Serum biochemical parameters					
Triglycerides (mg dL^− 1^)	33.0	32.4	4.47	0.900	0.114
Total cholesterol (mg dL^− 1^)	72.2	76.9	5.77	0.410	0.588
Liver enzymes					
Alanine aminotransferase (U L^− 1^)	17.3	16.9	1.53	0.824	0.143
Aspartate aminotransferase (U L^− 1^)	120	112	7.19	0.338	0.387
Protein profile					
Total protein (g dL^− 1^)	6.85	6.35	0.29	0.085	0.771
Albumin (g dL^− 1^)	5.76	5.90	0.26	0.582	0.737
Energy metabolism					
Glucose (mg dL^− 1^)	107	114	5.16	0.222	0.344
Renal function					
Urea (mg dL^− 1^)	64.8a	56.0b	4.22	0.035*	0.314
Creatinine (mg dL^− 1^)	0.76	0.71	0.02	0.236	0.010*

Values are presented as least squares means ± standard error of the mean (SEM)

*p*-values refer to the fixed effect of treatment

*p*-value_i_ refers to the effect of the initial value included as a covariate in the model

Statistical significance was set at *p* ≤ 0.05 (*) and *p* ≤ 0.01 (**)

### Serum biochemical parameters

Dietary treatment did not influence triglycerides, total cholesterol, ALT, AST, total proteins, albumin, glucose, or creatinine (*p* > 0.05; Table 1). However, serum urea concentration was higher in DDGS diet compared to the control diet (64.8 vs. 56.0 mg/dL; *p* = 0.035). The initial value included as a covariate significantly influenced hemoglobin (*p* = 0.049), packed cell volume (*p* = 0.027), mean corpuscular hemoglobin concentration (*p* = 0.025), leukocytes (*p* = 0.003), neutrophils (*p* = 0.005), and creatinine (*p* = 0.010).

## Discussion

The inclusion of agro-industrial by-products in ruminant diets has been widely investigated as a strategy to reduce feeding costs and improve the sustainability of intensive production systems. In the present study, DDGS inclusion in diet did not affect growth performance, carcass traits, or feed efficiency of feedlot lambs. These results indicate that DDGS can replace conventional protein sources in finishing diets without compromising productive performance. This pattern has also been reported in cattle (Brunetto et al. 2025) and sheep (Reddy et al. 2024). In addition, DDGS has been recognized as a cost-competitive alternative feed ingredient in several production systems (Brunetto et al. 2025). However, because no economic evaluation was performed in the present study, no conclusions regarding feed cost reduction or profitability can be drawn from these results.

The absence of treatment effects on ADG, FBW, and BCS suggests that the nutritional value of the DDGS-based diet was comparable to that of the control diet. DDGS is recognized as a nutrient-dense ingredient, containing high levels of protein, digestible fiber, and fat, which can support animal growth when properly balanced in the diet (Virgínio Júnior et al. 2025). The use of a 100% concentrate diet reflects intensive feedlot systems commonly adopted during the finishing phase of lamb production. Because such diets may increase the risk of subacute ruminal acidosis and other digestive disturbances, animals were gradually adapted over a 21-day period before the beginning of the experimental phase. Although ruminal fermentation characteristics and rumen morphology were not evaluated in the present study, the absence of reductions in productive performance or alterations in key metabolic indicators suggests that the dietary adaptation protocol was effective in maintaining normal physiological function under the conditions evaluated. Therefore, conclusions regarding ruminal health should be interpreted with caution and confirmed in future studies including direct measurements of rumen function.

Previous studies have also reported similar growth performance between lambs fed DDGS and those receiving conventional protein sources (Quadros and Kerth 2022; Thompson et al. 2024), indicating that moderate inclusion levels generally do not impair productive responses. For instance, Reddy et al. (2024) observed no differences in weight gain or feed efficiency in lambs supplemented with DDGS. Similarly, Chelkapally et al. (2023), in a meta-analysis, concluded that DDGS can be safely incorporated into sheep diets without negatively affecting performance when included at moderate levels.

The lambs fed the DDGS diet showed lower DMI relative to BW compared those on the control diet. This intake reduction may be associated with the higher nutrient density of DDGS-based diets (Avila-Stagno et al. 2013), specifically their elevated protein and lipid concentrations. Furthermore, dietary palatability might have played a role, as the distinct sensorial characteristics of DDGS can occasionally limit voluntary consumption. A similar response was clearly observed by Sorensen et al. (2021), who reported an immediate decrease in intake as DDGS inclusion levels increased. Diets with higher energy density may promote earlier satiety, thereby reducing voluntary intake without compromising weight gain. The higher serum urea level, coupled with the trend toward higher plasma protein levels, indicates that the DDGS diet provided greater protein availability than control. This supports the mechanism of metabolic satiety regulating feed intake. Similar findings have been reported in ruminants fed DDGS-based diets where improved nutrient utilization efficiency offset reductions in intake (dos Santos et al. 2024). The maintenance of ADG despite the lower DMI relative to body weight suggests that DDGS did not impair animal performance. However, because feed efficiency (FE) and feed conversion ratio (FCR) were not affected by treatment, no improvement in feed utilization efficiency can be inferred from these results.

The lack of treatment effects in carcass traits, including LEA and BT, indicates that DDGS inclusion did not alter patterns of muscle development or fat deposition during the finishing period. Carcass composition in lambs is strongly influenced by energy intake and overall growth rate, and the similar performance observed between treatments likely contributed to the comparable carcass characteristics (Zhang et al. 2023). Previous research evaluating DDGS supplementation in small ruminants has produced variable results depending on inclusion level, diet composition, and feeding system (Ale et al. 2022; Quadros and Kerth 2022; Chelkapally et al. 2023; dos Santos et al. 2024). However, several studies have reported minimal or no changes in carcass traits when DDGS replaces traditional protein ingredients at moderate levels, supporting the findings observed in the present study.

From a health perspective, parasitological indicators and anemia-related parameters were not affected by dietary treatment. EPG, Famacha© score, and PCV remained similar between treatments throughout the experimental period, indicating that DDGS inclusion did not influence fecal egg shedding or anemia risk. The association of these variables is considered an excellent indicator of animal health status (Ferreira et al. 2019), and the results of this study suggest that the nutritional modifications introduced by DDGS did not markedly alter host resistance or resilience to gastrointestinal parasites under the conditions evaluated.

Most studies evaluating DDGS supplementation in lamb diets have been conducted under grazing conditions, and similarly reported no effect on parasite infection (Tafernaberry et al. 2019). The variation observed over time for EPG and PCV likely reflects natural fluctuations in parasite dynamics rather than dietary effects. In addition, the pen environment, together with the low initial parasite infection and the provision of a balanced diet, may have contributed to preventing the progression of the mild natural infection by *Haemonchus* spp. observed in lambs from both treatments. The Famacha© score improved (decreased), and PCV increased throughout the trial, reinforcing the progressive healthy condition of the lambs.

Regarding hematological parameters, most erythrogram variables were not affected by dietary treatment, indicating that DDGS inclusion did not impair erythropoiesis or oxygen transport capacity. Values for red blood cells, hemoglobin, and packed cell volume remained within the physiological ranges reported for sheep (Seixas et al. 2021), reinforcing the conclusion that the experimental diets maintained adequate nutritional and health status.

Lambs fed the DDGS-based diet exhibited lower total leukocyte and neutrophil counts compared with animals receiving the control diet. In contrast, Gite et al. (2024), evaluating higher inclusion levels of DDGS, reported increased leukocyte counts in Nagavali ram lambs, suggesting that hematological responses may vary according to the level of DDGS included in the diet. Although leukocytes play a key role in immune function, the values observed in both treatments remained within the physiological ranges reported for sheep (Seixas et al. 2021). Moreover, the absence of differences in parasitological indicators or clinical signs indicates that the lower leukocyte and neutrophil counts were not associated with detectable adverse health effects under the conditions of the present study. Because inflammatory biomarkers were not evaluated, these hematological differences should not be interpreted as evidence of reduced inflammatory status or improved immune function.

Serum biochemical parameters also indicated that DDGS inclusion did not negatively affect metabolic health. Lipid profile variables, including triglycerides and cholesterol, as well as liver enzymes such as ALT and AST, were not influenced by dietary treatment, suggesting that lipid metabolism and hepatic function remained stable (Reece and Swenson 2004). Similarly, indicators related to energy metabolism (glucose) and protein status (total proteins and albumin) were unaffected by DDGS inclusion, demonstrating that the experimental diets provided adequate metabolic support for lambs, such as study of Gite et al. (2024) in lambs and Ale et al. (2022) in male young goats. These findings are particularly relevant because excessive dietary nutrients or metabolic by-products can impose physiological overload on detoxification and excretory organs, especially the liver and kidneys (Van Saun 2023). In ruminants, the liver plays a central role in nutrient metabolism and detoxification, while the kidneys are responsible for the excretion of nitrogenous wastes and other metabolites. When metabolite concentrations increase beyond the organism’s metabolic capacity, these organs may experience functional stress, often reflected by alterations in serum biochemical markers. However, the stability of hepatic enzymes and protein-related indicators observed in the present study suggests that DDGS inclusion did not lead to excessive metabolite accumulation or metabolic overload, indicating that hepatic processing and renal excretion remained within normal physiological limits.

The only biochemical variable affected by treatment was serum urea concentration, which was higher in lambs receiving the DDGS diet. Elevated serum urea levels are commonly associated with increased dietary protein intake or enhanced ruminal protein degradation, leading to greater ammonia production and subsequent conversion to urea in the liver (Reece and Swenson 2004). Considering that the DDGS-based diet presented higher crude protein content than the control diet, the increase in serum urea likely reflects greater nitrogen intake rather than impaired renal function or metabolic imbalance. Importantly, creatinine concentrations were not affected by treatment, indicating that kidney function remained normal.

Overall, the results of this study demonstrate that moderate inclusion of DDGS can be used in feedlot lamb diets without detrimental effects on performance, carcass development, or metabolic health. These findings support the growing body of evidence (Tafernaberry et al. 2019; Reddy et al. 2021, 2024; Quadros and Kerth 2022; dos Santos et al. 2024; Gite et al. 2024; Thompson et al. 2024) indicating that ethanol industry by-products represent viable alternative feed ingredients for small ruminant production systems, particularly in regions where feed costs represent a major constraint for intensive finishing operations.

## Conclusion

DDGS inclusion in sheep effectively maintains growth performance and carcass characteristics in feedlot lambs. The observed reduction in relative DMI without compromising ADG indicates that DDGS maintained animal performance under the conditions of this study. However, because feed efficiency and feed conversion ratio were not affected, no improvement in feed utilization efficiency can be concluded. The higher serum urea concentration observed in DDGS-fed lambs likely reflects increased dietary protein intake and metabolic availability. Furthermore, the preservation of hematological and biochemical profiles confirms that DDGS is a safe alternative protein source that did not adversely affect the evaluated hematological and serum biochemical indicators in intensive sheep production systems.

## Data Availability

Not applicable.
